# Spontaneous Rupture of the Gravid Uterus in a Postmenopausal Woman Receiving Fertility Treatment

**DOI:** 10.7759/cureus.5132

**Published:** 2019-07-13

**Authors:** Frank Yuan, Sindy H Wei, Johnathan Chen

**Affiliations:** 1 Radiology, David Geffen School of Medicine at University of California Los Angeles (UCLA) / Ronald Reagan Medical Center, Los Angeles, USA; 2 Radiology / Abdominal Imaging, David Geffen School of Medicine at University of California Los Angeles (UCLA) / Ronald Reagan Medical Center, Los Angeles, USA; 3 Radiology / Pediatric Imaging, David Geffen School of Medicine at University of California Los Angeles (UCLA) / Ronald Reagan Medical Center, Los Angeles, USA

**Keywords:** uterine rupture, fertility treatment, in vitro fertilization, obstetric emergency, assisted reproductive technology, postmenopausal women

## Abstract

Uterine rupture is an uncommon obstetric emergency that is potentially fatal to the mother and fetus. Spontaneous rupture of the unscarred gravid uterus in postmenopausal women who achieve pregnancy through in vitro fertilization (IVF) has been infrequently described in the literature. We present the case of a 72-year-old postmenopausal woman, gravida 1 para 0, who conceived by donor oocyte IVF in Europe and subsequently suffered uterine rupture at 22 weeks gestation with large hemoperitoneum. The patient underwent emergent laparotomy, with successful repair of the uterine wall defects. Postmenopausal women face an increased risk of spontaneous uterine rupture and life-threatening bleeding, which is likely due to uterine atrophy and limited uterine capacity. Further research is needed to establish age-appropriate guidelines for selecting treatment candidates.

## Introduction

Spontaneous uterine rupture is an uncommon but often fatal obstetric complication related to pregnancy. There is a paucity of literature discussing uterine rupture of the unscarred uterus following in vitro fertilization (IVF), particularly for post-menopausal patients in advanced age. Recent population studies have demonstrated a growing demand of older women seeking infertility treatments such as IVF [1]. In a post-menopausal patient with a significantly atrophic uterus, IVF may put the patient at increased risk for rupture and life-threatening hemorrhage. We present the case of a woman that was treated for infertility who developed spontaneous uterine rupture.

## Case presentation

A 72-year-old post-menopausal woman, gravida 1 para 0 at 22 weeks gestation, presented to our institution with complaints of nausea, vomiting, and abdominal pain. The patient’s obstetric and gynecologic history included oocyte donation and in vitro fertilization (IVF) using donor sperm in Europe. After treatment, she returned to the United States where she resided and remained asymptomatic until her presentation. The rest of her past medical history was only remarkable for a prior laparoscopic appendectomy for appendicitis.

**Figure 1:**
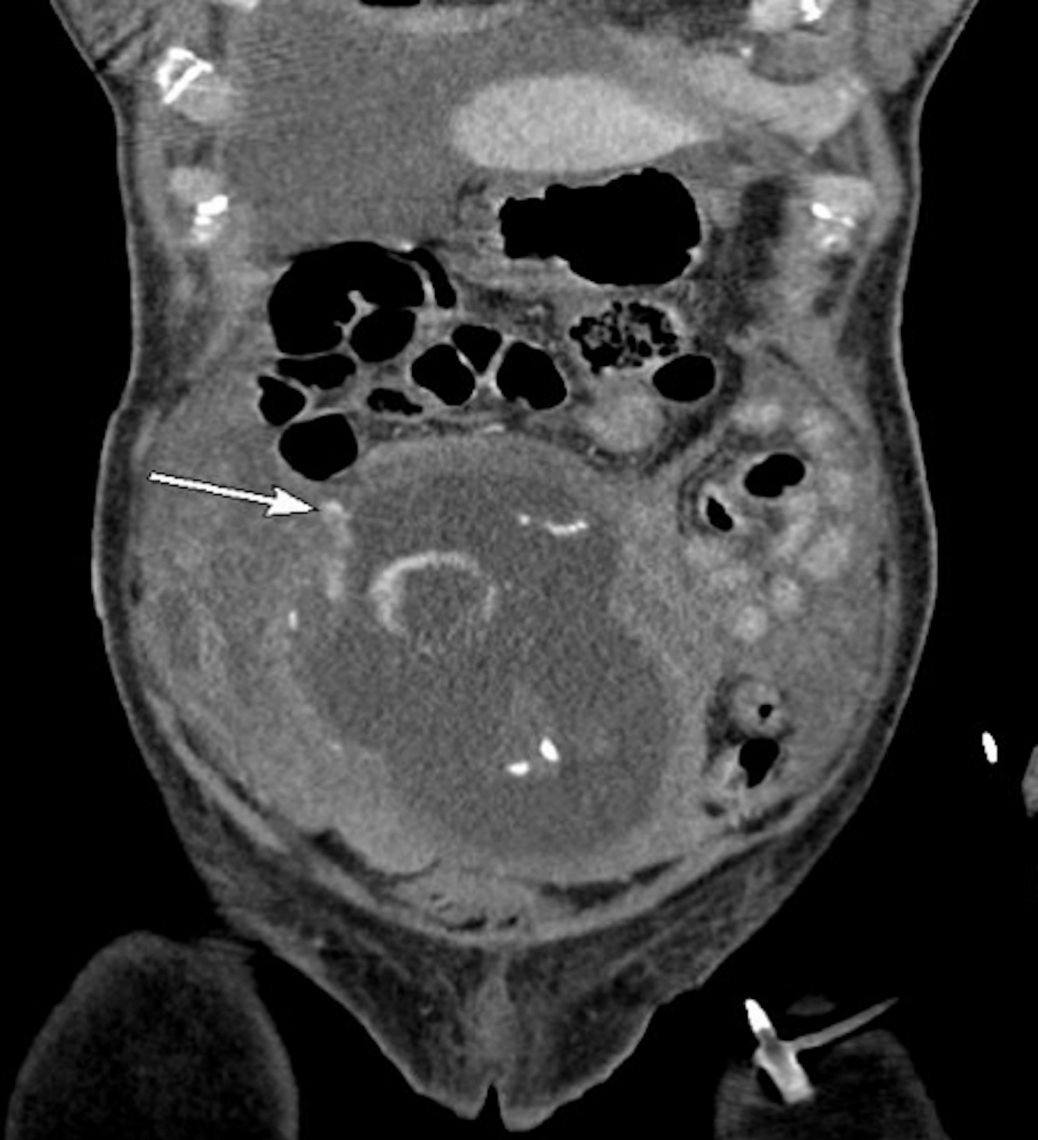
Computed tomography of the abdomen and pelvis. Coronal view showing active radiodense contrast extravasation from the perforated focal area of thinning of the right anterolateral fundus where there is a contrast blush (arrow).

**Figure 2:**
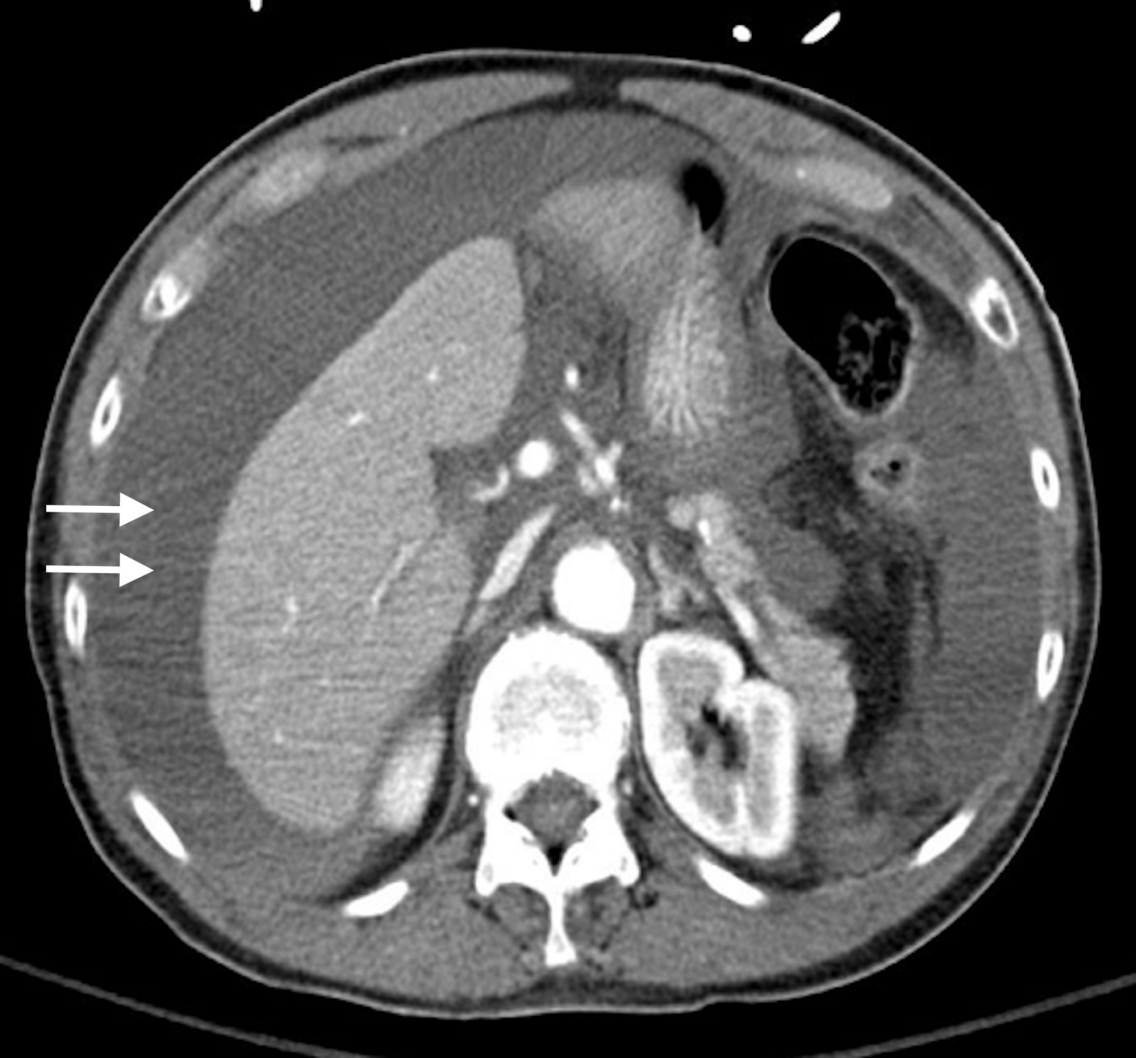
Computed tomography of the abdomen and pelvis. Axial view showing moderate to large amount of low attenuation fluid in the peritoneum, consistent with hemoperitoneum.

**Figure 3:**
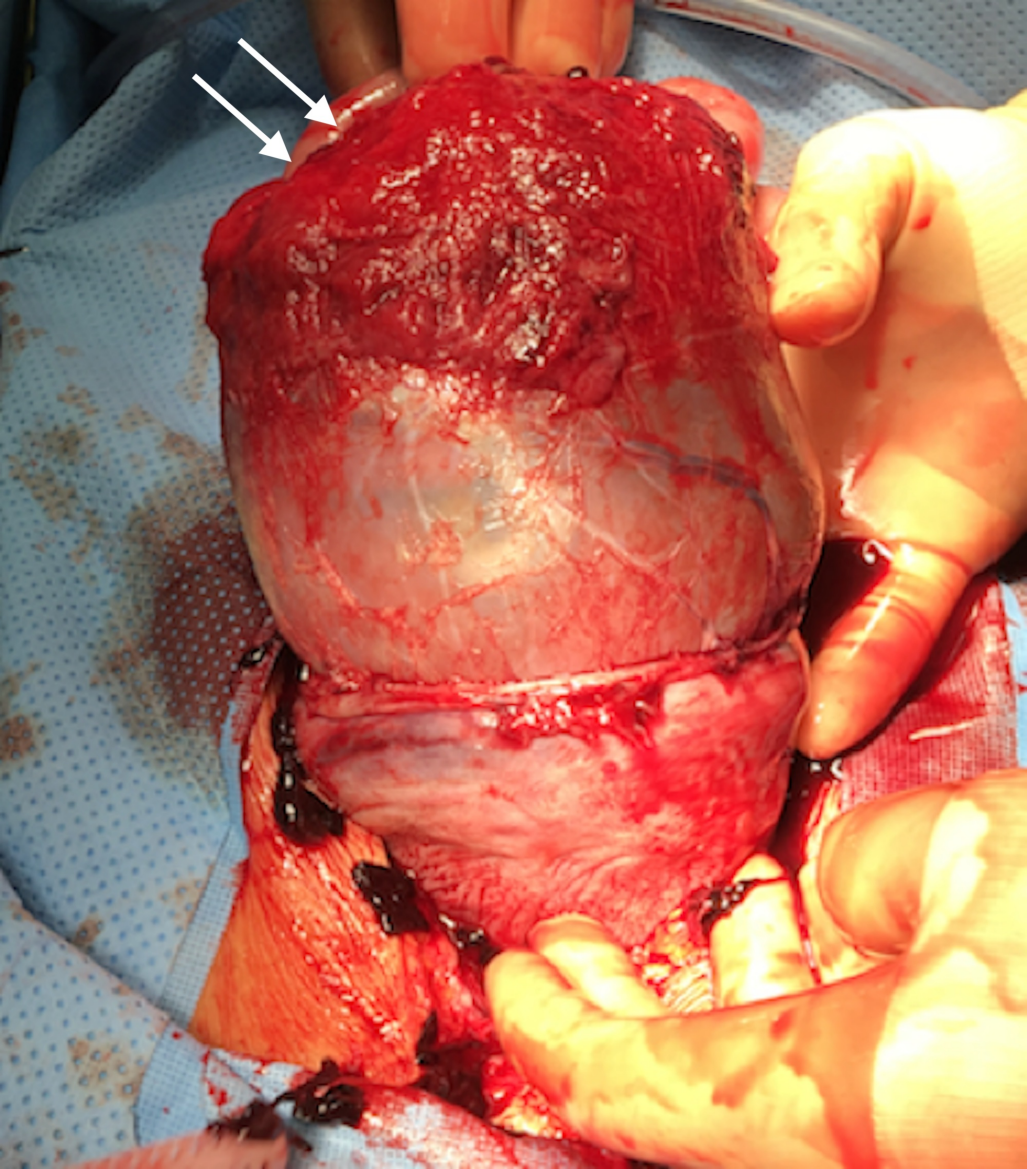
Intraoperative photograph of the perforated uterus (arrow) at the fundus.

**Figure 4:**
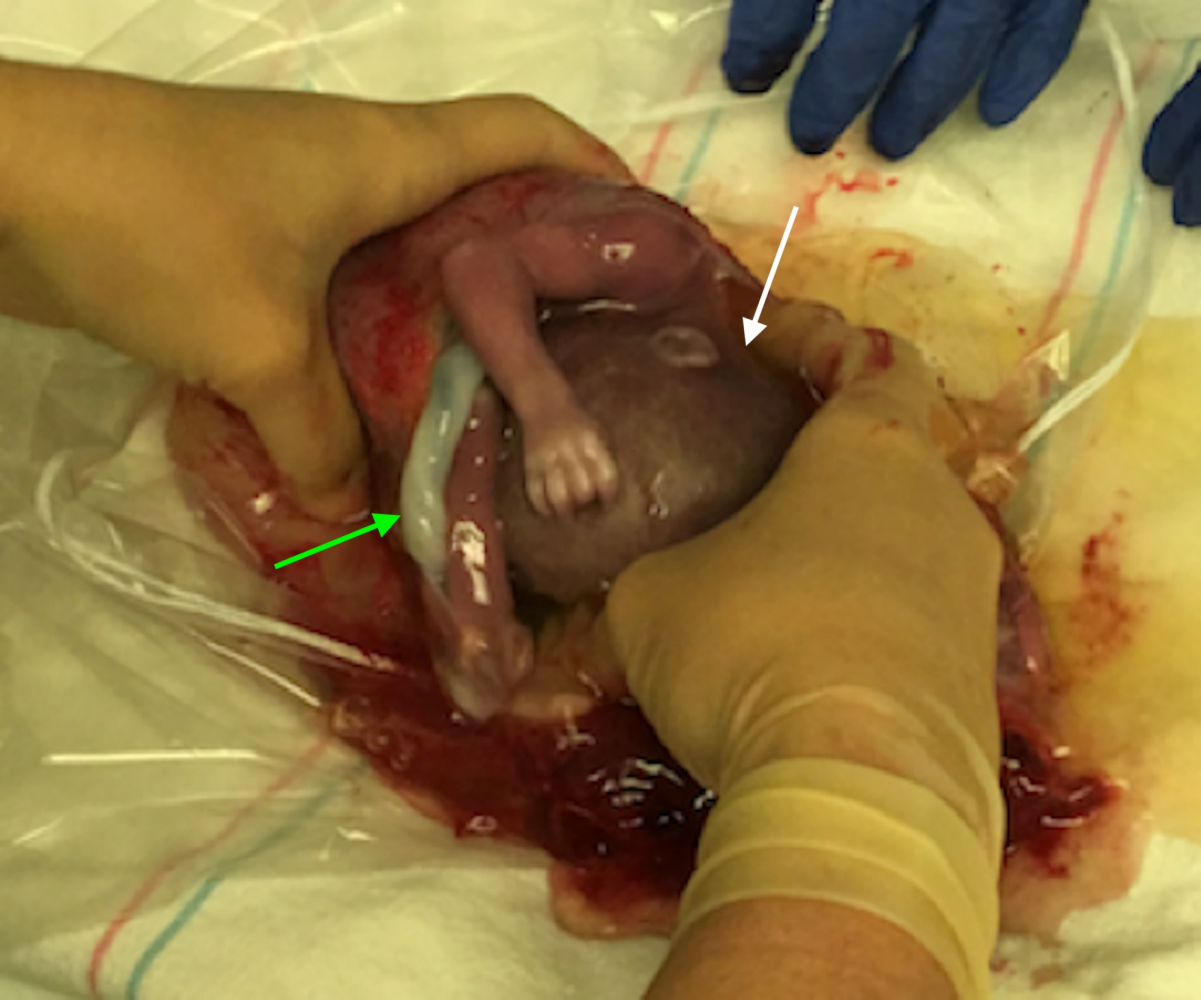
Intraoperative photograph of the intact stillborn fetus (white arrow) in the sac along with the placenta (green arrow) being delivered.

Shortly after her arrival, the patient’s blood pressure decompensated to a low of 85/50 mm Hg in the emergency department. A contrast-enhanced computed tomography (CT) was performed which showed fundal discontinuity and active extravasation of contrast into the peritoneum (Figures 1, 2). Additionally, her scan showed a large amount of hemoperitoneum confirming the diagnosis of spontaneous uterine rupture. The fetus was within the endometrial cavity at the time CT was performed; however, fetal heart tracing did not show any signs of activity. The patient was urgently transferred to the operating room for an emergent laparotomy. At the time of operation, the fetus and gestational sac were partially extruded from the defect at the uterine fundus seen on CT (Figures 2, 3). The fetus, sac, and placenta were delivered and handed to the pediatricians intact (Figures 3, 4). Subsequently, the fetus was found to be stillborn. There was no active uterine bleeding, so the decision was made not to perform a hysterectomy. A primary repair of the ruptured uterus was performed with 0 Monocryl to achieve adequate hemostasis. The patient’s bowel was inspected, all adhesions were lysed, and her abdomen was irrigated with warm saline. Approximately 3.5 L of estimated blood loss into the peritoneum was evacuated. The laparotomy incision was then closed.

## Discussion

Uterine rupture has a high rate of maternal mortality, reported up to 13.5% in one single center series. The mortality for the fetus is even higher, reported up to 89.1% as a result of intrauterine death. When compared to a previously scarred uterus, rupture of the unscarred uterus carries higher risks for both the mother as well as the fetus [2]. Fortunately, the incidence of uterine rupture in developed countries has been low in both women with a scarred uterus (0.5%) and unscarred uterus (0.08%) [3].

However, data from a recent population-based study demonstrated a trend of increasing demand for assisted reproductive technology (ART) by older women [1]. In the United States, there has been an increase in demand for IVF, with the most significant increase in the number of treatments of 14% (7,157 in 2012 to 7,495 in 2013) in those aged 45-49 years [4]. Although IVF has demonstrated promising efficacy as an infertility treatment, in patients who are postmenopausal, success rates are variable and are not without serious risk [5]. In oocyte recipients, complications such as pregnancy-induced hypertension, preeclampsia, gestational diabetes, preterm labor, and increased cesarean section have been commonly reported [6]. One potential complication in very advanced age patients is uterine rupture, presumably due to atrophy of the native uterus from reduced hormone stimulation [7].

Spontaneous uterine rupture during the first and second trimester has been reported after IVF and can lead to life-threatening maternal hemorrhage [5]. Reports of women who developed uterine rupture following infertility treatments are scarce [5, 8]. In particular, there is a paucity of literature describing spontaneous uterine rupture following IVF in postmenopausal patients with advanced age. Our review of PUBMED from inception to 2017 using “uterine rupture after in vitro fertilization,” “advanced maternal age in vitro fertilization uterine rupture,” and “pregnancy complications in vitro fertilization” revealed no similar cases to ours.

In this report, we presented such case of a 72-year-old postmenopausal woman who was treated outside of the United States with oocyte donation and IVF and suffered a rupture of the previously unscarred uterus at 22 weeks gestation. Commonly reported symptoms alarming an impending uterine rupture in women not undergoing active labor include hypotension, tachycardia, vaginal bleeding, severe abdominal pain and tenderness, hematuria, and postpartum hemorrhage [2]. Our patient presented with abdominal distention, along with nausea and vomiting, that rapidly progressed into poorly localized pain and tenderness on exam. Hemodynamic instability noted in the ED suggested active extravasation from the perforated site. She was transferred for urgent exploratory laparotomy and operative management. The absence of fetal heart tones or fetal distress may also provide clues to an impending rupture. Common risk factors for uterine rupture include a history of uterine and tubal surgeries, the most common being cesarean section, placental implantation abnormalities, and uterine malformations [8]. Our patient did not have any of the precipitating factors mentioned above. Focal and generalized myometrial thinning seen on CT is likely related to uterine atrophy in the postmenopausal state and is likely a key precipitating factor. The uterus begins to grow at the onset of puberty, and its size fluctuates with the menstrual cycle in premenopausal women. In adulthood, variation in uterine size is primarily related to a woman’s parity and is not significantly affected by her age. After the menopause, the uterine volume, size, and the corpus-cervix ratio begin to decrease progressively. The reduction in uterine size has been related to years after menopause, presumably from the lack of hormone stimulation and decreased uterine blood flow [9]. Since our patient has been in the postmenopausal state for two decades, atrophy of the uterine wall and a reduced uterine cavity capacity likely contributed to her complication.

Despite a growing demand for infertility services, women older than the age of 40 have limited access to ART primarily due to the lack of insurance coverage - a product of age-dependent rationing of medical care [1]. Older patients seeking infertility care frequently require more treatments, experience less success, and face more risks. Therefore, the cost-effectiveness of IVF decreases with advancing maternal age [1, 6]. Cost-effectiveness remains the principal argument against providing coverage for older patients, and in most developed countries, advanced female age is considered a definite barrier to treatment. Globally, the cost-effectiveness of infertility care defined by individual countries is always linked to the country’s unique geopolitical atmosphere and with economic considerations. For example, countries like Israel provides government-subsidized IVF in response to its declining population growth while other countries offer little to no subsidy. The greatly varying methods in how cost-effectiveness is assessed in different countries further complicate the development of universal guidelines. In the United States (US), regulations of ART exist at three levels: federal agencies, state legislation, and professional societies. Federal agencies such as the Center for Disease Control (CDC) and the Food and Drug Administration (FDA) provide oversight to ensure accurate data reporting and appropriate reproductive tissue handling. The American Society of Reproductive Medicine (ASRM) is the main governing body in the US that provide practicing guidelines at the individual level. A considerable inter-state variation in laws relating to health insurance coverage for infertility care exists. Currently, only 15 states have passed laws that require insurers to either cover or offer coverage for infertility treatments in the US. When facing older patients willing to pay a sizeable out-of-the-pocket fee for infertility treatments, some practitioners may offer regimens that are more aggressive than the standard dosages to offset the anticipated poorer outcomes [1]. Older patients may not tolerate repeated cycles of aggressive hormone stimulation and are more likely to experience life-threatening complications. The patient we presented serves as an example of inappropriate patient selection. We currently do not have evidence-based guidelines to assess risks related to women over the age of 50 undergoing oocyte donation and IVF. With a growing demand for assisted reproductive technology (AST), further research is needed to establish age-appropriate guidelines to ensure patient safety.

## Conclusions

Postmenopausal women face an increased risk of spontaneous uterine rupture and life-threatening bleeding, which is likely due to uterine atrophy and limited uterine capacity. Further research is needed to establish age-appropriate guidelines for selecting treatment candidates.
